# Diagnostic and Therapeutic Insights into Spinal Glomangioma of a Unique Intradural, Extramedullary Presentation—Systematic Review

**DOI:** 10.3390/diseases12060132

**Published:** 2024-06-20

**Authors:** Wojciech Czyżewski, Jakub Litak, Barbara Pasierb, Paula Piątek, Michał Turek, Lech Banach, Grzegorz Turek, Kamil Torres, Grzegorz Staśkiewicz

**Affiliations:** 1 Department of Neurosurgery, Maria Sklodowska-Curie National Research Institute of Oncology, ul. W.K. 7 Roentgena 5, 02-781 Warsaw, Poland; 2 Department of Didactics and Medical Simulation, Medical University of Lublin, 20-954 Lublin, Poland; 3 Department of Clinical Immunology, Medical University of Lublin, 20-954 Lublin, Poland; jakub.litak@gmail.com; 4 Department of Dermatology, Radom Specialist Hospital, Lekarska 4, 26-600 Radom, Poland; barbarapasierb95@gmail.com; 5 Department of Neurosurgery, Medical College of Rzeszow University, 35-959 Rzeszow, Poland; paulapiatek89@gmail.com; 6 Department of Neurosurgery, Postgraduate Medical Centre, Brodnowski Masovian Hospital, 8 Kondratowicza Str., 03-242 Warsaw, Poland; michal.turek1997@gmail.com (M.T.); turek.grz@gmail.com (G.T.); 7 Alfamed Pathomorphology Department, 22-400 Zamosc, Poland; lechbanach@gmail.com; 8 Department of Plastic, Reconstructive Surgery with Microsurgery, Medical University of Lublin, 20-954 Lublin, Poland; kamiltorres@wp.pl; 9 Department of Human, Clinical and Radiological Anatomy, Medical University, 20-954 Lublin, Poland; grzegorz.staskiewicz@gmail.com

**Keywords:** glomangioma, glomus tumor, spinal tumor, intradural extramedullary

## Abstract

Contemporary literature lacks examples of intradural, extramedullary spinal glomangiomas. Moreover, glomus tumors in general are exceedingly rare among benign spinal tumors and are mostly located within epidural space or within intervertebral foramen, and only a few cases have been documented to date. This report provides a detailed analysis of the clinical presentation, imaging characteristics, surgical intervention, and pathological findings of a 45-year-old patient experiencing progressive locomotor deterioration. The tumor was surgically excised, and subsequent histological examination identified it as a representative of glomus tumors—a glomangioma. Notably, this represents a unique case as it was the first example of such a tumor being discovered intradurally. Radical surgical excision remains the modality of choice in most benign spinal tumors of this localization. Although the malignant transformation of glomus tumors within the spine has not been documented thus far, cases have arisen in other areas. Consequently, we will investigate potential oncological treatments for cases with malignant potential and highlight advancements in surgical techniques for benign intradural spinal tumors.

## 1. Introduction

Spinal tumors comprise around 15% of central nervous system tumors [1]. They are categorized as primary benign or primary/metastatic malignant tumors. While benign lesions make up to 80% of all primary spinal cord tumors, the remainder are malignant [2]. Anatomical localization within the spinal column distinguishes them as intradural or extradural. Intradural tumors can be further classified based on their relationship to the spinal cord as either intramedullary (IDIM) or extramedullary (IDEM). Primary spinal neoplasms exhibit a relatively low incidence rate, accounting for approximately 2–4% of CNS tumors, with an estimated 65% manifesting as intradural extramedullary lesions [3,4].

Although the manifestation of intradural extramedullary tumors may not invariably demonstrate pathognomonic traits and their presentation is most commonly related to the affected spinal cord level, the utilization of magnetic resonance imaging (MRI) frequently facilitates the recognition of essential characteristic features. Given the anatomical proximity to spinal nerves or the dura mater, schwannomas, meningiomas, and ependymomas emerge as the predominant primary tumors of the spinal canal [5,6]. Certain malignant primary [7,8,9,10] or metastatic [11,12] intradural lesions, although infrequent, may resemble their primary counterparts; however, their initial management differs as it typically requires adjunct oncological treatment [13,14]. Among the less commonly encountered non-malignant tumors are ependymomas, hemangiomas, lipomas, paragangliomas, vascular neoplasms, nerve sheath myxoma, and other exceptionally rare entities, including the seldom-described glomus tumors of the spinal canal [15,16].

In this study, we present a case report elucidating a unique example of an intradural extramedullary histological variant of a glomus tumor—glomangioma.

## 2. General Information and Histopathological Considerations

Glomus tumors, categorized as mesenchymal tumors in the latest WHO Classification of Tumors (2021), account for less than 2% of soft tissue neoplasms [17,18]. In contrast to paragangliomas, which stem from chromaffin cells [19], glomus tumors are thought to arise from the Sucquet–Hoyer canal situated within the glomus body—a specialized arteriovenous anastomosis regulating skin temperature. These structures are surrounded by layers of epithelioid glomus cells that express smooth muscle actin (SMA) [20,21]. Low temperatures prompt the relaxation of glomus cells, facilitating the opening of the anastomosis and redirecting blood flow away from the capillary network, thus conserving body heat [22]. Although glomus tumors typically manifest in the dermis or subcutis of the distal extremities, especially phalanges, a plethora of various other extracutaneous localizations has been described in the literature [23]. One conceivable rationale of this ever-present distribution is that these tumors may originate from abundant perivascular cells capable of differentiating into glomoid cells [23]. This can be hypothesized through the neural crest origin of glomus cells and the ability of perivascular cells to adopt neural characteristics [24,25]. Perivascular cells, or pericytes, are cells situated along the walls of blood vessels, originating primarily from mesenchymal stem cells during embryonic development from the mesoderm, the middle layer of embryonic germ cells [26,27]. In certain organs, such as the central nervous system, pericytes may also arise from neuroectodermal cells [28,29]. In adults, these cells can differentiate from local precursor cells or stem cells, including those in the bone marrow, and are capable of transforming into various cell types like chondrocytes, osteoblasts, and adipocytes. The specific origin of pericytes can vary by organ, and their migration and localization are critical during vascular development, where they embed in vessel walls to regulate blood flow, vascular stability, and angiogenesis [30].

While the precise etiology of glomus tumors remains elusive, several predisposing factors have been proposed to date. These include sporadic mutations in the glomulin gene (GLMN—coded by 1p21–1p22) and a diagnosis of neurofibromatosis type 1 [31,32]. Yanai et al. highlighted an association between neurofibromatosis type 1 (NF1) and glomangioma, with multiple glomus tumors characteristic of NF1 patients [33]. Brems et al. identified germline and somatic NF1 mutations, as well as RAS-MAPK hyperactivation, in NF1-associated glomus tumors, distinguishing them from sporadic cases [34].

Preliminary research has indicated that genetic modifications involve BRAF, NOTCH, PDGFRB, KRAS, and SMARCB1 [18,35,36,37,38]. Moreover, certain authors propose a traumatic etiology, drawing from a documented case involving a digital glomangioma [39]. Glomus tumors tend to affect individuals between 20 to 40 years old with female preponderance [23,40].

Glomus tumors are distinguished by their histological composition, primarily consisting of glomus cells, blood vessels, and vascular smooth muscle cells [23,41,42]. Within this tumor group, classification into three variants based on histological structure is feasible: solid tumors, glomangiomas, and glomangiomyomas [43,44,45] (Table 1). These variants display varying proportions of the aforementioned histological elements [42,43]. Hematoxylin and eosin (H&E) staining is conventionally employed for visualization [46,47,48]. Glomus cells are a consistent finding across all variants, with solid tumors predominantly composed of glomus cells, glomangiomas characterized by an abundance of blood vessels, and glomangiomyomas exhibiting a prevalence of smooth muscle cells among a network of blood vessels [41].

The microscopic examination of glomangiomas reveals vascular structures reminiscent of cavernous hemangiomas surrounded by round or oval cells featuring well-defined margins, eosinophilic cytoplasm, centrally located nuclei, and an absence of atypia [18,46,47]. Nevertheless, immunohistochemical analysis plays a pivotal role in confirming the diagnosis. Integrating standard histological examination with immunohistochemistry enhances diagnostic precision [49]. Several markers associated with glomus tumors aid in their identification, including smooth muscle actin (SMA), collagen type IV, vimentin, and muscle-specific actin (MSA) [50]. Conversely, glomus tumors typically lack positive staining for antibodies targeting S100, chromogranin, desmin, cytokeratin, HMB-45, melan-A, or synaptophysin [18,51]. The proliferation index, as indicated by Ki-67 staining, generally remains low. Furthermore, while tumor cells may exhibit positive staining for CD34, caldesmon, or calponin, there have been reports of negative immunostaining for these markers in selected cases [41]. Notably, positive staining for SMA serves as a definitive marker for distinguishing glomus tumors from other types of neoplasms [45]. 

Malignant glomus tumors are extremely uncommon, accounting for up to 2.9% of all glomus tumors, and are exclusively documented in the literature as isolated case reports originating from different institutions. Glomus tumors of uncertain malignant potential are defined by tumors that do not meet the standards for malignancy yet display at least one unusual feature other than nuclear pleomorphism [52].

The primary aim of this investigation was to outline both the diagnostic methodologies and treatment modalities for glomangiomas of the spinal canal. Furthermore, we conducted a systematic review of the literature to provide a comprehensive scientific understanding of this topic. Given that the standard approach for most benign intradural tumors typically involves radical excision, we also expounded upon various surgical techniques, including microsurgical, endoscopic, or robotic approaches. In certain instances where residual tumor remains due to incomplete resection or in cases where malignant potential necessitates adjuvant therapy, we discussed the current radiotherapy methods, as well as the employment of chemotherapy and immunotherapy for malignant cases.

## 3. Case Presentation

A 45-year-old male sought medical attention at our outpatient facility, reporting a progressive decline in locomotor function. Upon evaluation via magnetic resonance imaging (MRI), an oval-shaped intradural lesion was identified at the L3 vertebral level, occupying the spinal canal and exerting bilateral nerve root compression. Neurological examination revealed diminished lower extremity tendon reflexes and mild weakness in knee extension and hip external rotation, devoid of sensory deficits.

**Figure 1:**
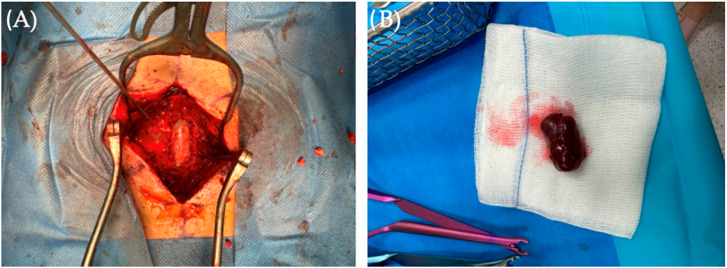
(**A**) Following laminectomy for spinal canal decompression, a bulging dural sack appeared with tumor located intradurally. (**B**) Picture presents the brownish-in-color tumor, resected en bloc.

Given the diagnostic findings, surgical intervention was deemed necessary. We employed a traditional microsurgical approach for radical tumor excision. In June 2023, the patient underwent L2, L3, and L4 laminectomy with intraoperative peripheral nerve function monitoring conducted by a neurophysiologist. Intraoperatively, a bulging of the dural sac at the L3 level was observed (Figure 1A). After precise incision and dissection, we performed dural splitting and microsurgical resection of the inner dura while preserving the outer layer using the Saito method. This exposed the arachnoid membrane, which was subsequently incised, revealing clear cerebrospinal fluid (CSF) and a solid, red-grayish tumor compressing the spinal nerve roots dorsally. The tumor had a small vascular peduncle, which was coagulated and cut. Careful microsurgical dissection enabled complete tumor removal, confirming its non-neural and non-dural origin (Figure 1B). Importantly, neither motor nor sensory responses (MEP and SEP) were decreased during or after surgery

**Figure 2:**
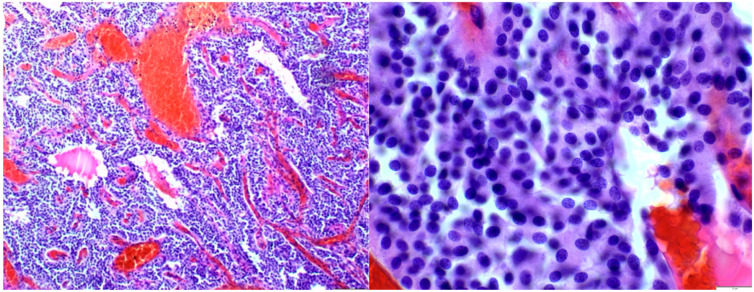
Histological analysis showed cohesive round cell proliferation around blood vessels with perivascular hyalinization.

Histological examination revealed a solid and syncytial proliferation of round cells surrounding blood vessels exhibiting perivascular hyalinization. Immunohistochemical staining demonstrated positivity for smooth muscle actin (SMA) and negativity for epithelial membrane antigen (EMA), glial fibrillary acidic protein (GFAP), S100, and GATA3, consistent with a diagnosis of glomangioma (Figure 2).

Following the procedure, the patient demonstrated gradual improvement and was mobilized by a physiotherapist on postoperative day one. The gradual resolution of paresis occurred within several days of hospitalization, leading to the patient’s discharge in a stable condition. Only mild, typical postoperative pain symptoms in the operated area remained. No CSF leakage was observed and the wound healed well.

**Figure 3:**
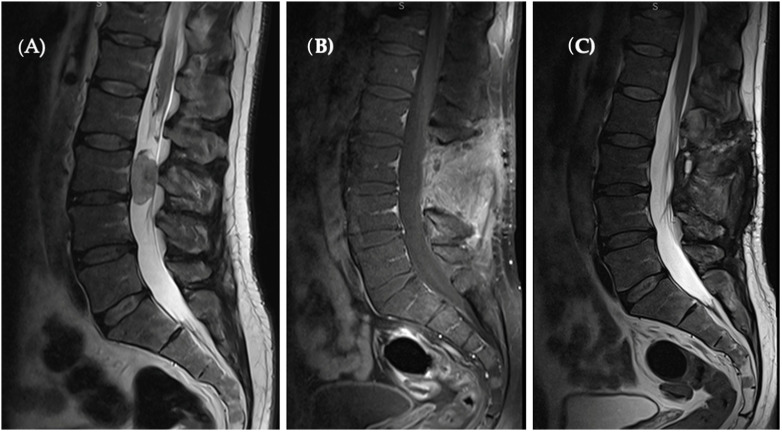
(**A**) Pre-op T2 sagittal MRI revealed a hyperintense oval-shaped lesion located at the L3 level occupying spinal canal. (**B**) Sagittal T1 with contrast enhanced as well as T2 sagittal. (**C**) Sections present a complete tumor removal and no sign of recurrence.

Subsequent MRI imaging undertaken after a one-year period displayed no evidence of tumor recurrence, precluding the necessity for adjuvant therapy (Figure 3).

## 4. Materials and Methods

**Figure 4:**
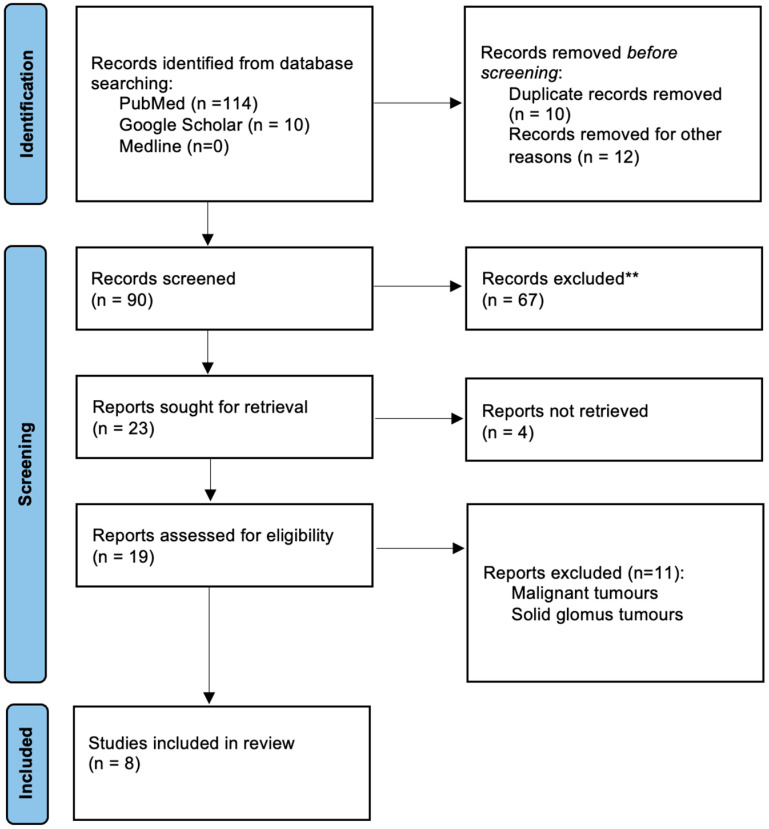
Flowchart of the identification and selection of studies according to the 2020 PRISMA statement.

The authors conducted a comprehensive systematic review following the Preferred Reporting Items for Systematic Reviews and Meta-Analyses (PRISMA) guidelines, ensuring meticulous examination of relevant literature. The search, conducted in March 2024, included studies published in English without time restrictions. Renowned biomedical databases such as PubMed, Medline, and Google Scholar were utilized, with two queries created: one for PubMed and Medline (“glomangioma” AND “spine” OR “spinal”), and another for Google Scholar (“glomus tumors” OR “glomangioma” AND “spine” OR “spinal” NOT “hand” NOT “foot”). Initially, 124 records were identified, but after screening and eligibility assessments, 19 studies addressing spinal glomus tumors were included. Two independent researchers performed each review step. Notably, the focus was primarily on glomangiomas of the spine, excluding malignant glomus tumors and other benign types. Ultimately, only eight publications met the stringent inclusion criteria and were used in synthesizing the findings of this study (Figure 4).

## 5. Results

Among the 19 reports identified in our systematic review that described cases of glomus tumors of the spine, only a limited number included cases of glomangioma, totaling eight instances. Key clinical characteristics extracted from these studies encompassed patient demographics (gender and age), tumor localization, neurological deficits, treatment modalities, time to diagnosis, malignant potential, recurrence rates, and additional therapeutic interventions when required. In the analyzed cases, there were four female and four male patients, with a median age of 48.6 years. The youngest patient was 26 and the oldest was 65 years old. Each patient underwent magnetic resonance imaging (MRI), revealing an intraspinal tumor mass, with six cases occupying intervertebral foramina and two located within the epidural space. Predominantly, tumors were situated at the thoracic level (five cases), but one was located on the cervico-thoracic junction with the remaining two in the cervical or lumbar regions. The most prevalent clinical presentation was chronic pain, while the remaining patients exhibited progressive paraparesis or an unsteady gait. The mean time to diagnosis circulates around 4 months. Surgical intervention alone was undertaken in most of the cases, except for one patient who received adjuvant therapy in the shape of radiotherapy. Among the surgically treated patients, none experienced recurrence during follow-up assessments, and histopathological examination did not reveal malignancy. Two patients exhibited uncertain malignancy through histopathology and only one required subsequent radiotherapy.

## 6. Discussion

To date, the literature records a total of 19 documented cases of glomus tumors associated with the spinal canal, among which only eight cases are specifically classified as glomangiomas (Table 2). The remaining tumors are solid glomus tumors, and there is one documented case of glomangiomyoma thus far [53]. In general, including solid glomus tumors, these lesions predominantly involve the thoracic level, followed by the lumbar spine, while cervical involvement is the least common, which is similar to the data obtained from the present systematic review on glomangiomas. Among glomangiomas of spinal origin, the intervertebral foramina remains the most common location followed by the epidural space. Despite the inclusion of case reports with systematic reviews encompassing glomus tumors as a collective entity, none have exclusively focused on glomangiomas. Additionally, variations in the reported number of cases are evident across the studies found. Notably, all glomangiomas referenced in the present review originate either from the epidural space or are situated within the intervertebral foramina. The case presented herein represents the sole example in the extant literature demonstrating an intradural location, thus posing diagnostic challenges due to their potential resemblance to more common spinal pathologies.

Generally, the cornerstone of treatment for all intradural, extramedullary lesions remain radical surgical excision, which typically results in favorable long-term outcomes with minimal risk of recurrence [54,55]. However, approximately 10% of patients experience a recurrence after surgery due to incomplete removal [44]. In instances where total resection is unattainable, observation may be considered for small, asymptomatic tumors. The most employed surgical technique involves traditional microscopic tumor excision, and the same proven tactics were used by authors of the study, which enabled reaching a favorable outcome. Nevertheless, this classical method entails making an extended midline incision (typically two levels above and below the tumor), dissecting the paraspinal muscles subperiosteally, and performing multi-level laminectomies or facetectomies. This approach damages a significant portion of the structures within the posterior column, leading to decreased spinal stability and the potential for spinal deformity [56]. Moreover, surgical treatment inherently poses risks, highlighted by ACS-NSQIP data on spinal tumor surgery outcomes. According to their research, key adverse outcome predictors include, e.g., dependent functional status, emergency surgeries, and higher ASA classifications. This underscores the importance of preoperative risk stratification and enhanced post-discharge surveillance [57].

Minimally invasive endoscopic techniques are increasingly adopted in modern medical practice for precise and effective spinal tumor removal by utilizing small bone windows and multi-angled endoscopes to access and excise lesions that are challenging under a microscope [58]. Early pioneers like Burtscher and subsequent practitioners like Barami and Caballero-García have documented the successful applications of these methods, reporting excellent clinical outcomes across various studies and approaches [59,60,61]. The novel unilateral biportal endoscopy (UBE) technique in minimally invasive spinal surgery enhances instrument precision and view and is increasingly used for tumor treatments due to its flexibility, minimal trauma, and reduced pain [62,63]. It was first successfully applied to intradural extramedullary tumor excision by Kujur et al. [64].

Although intraoperative monitoring is commonly used during spine tumor surgeries without confirmed effectiveness through prospective trials, Kujur and colleagues’ analysis of surgeries performed without intraoperative nerve monitoring (IONM) highlighted its potential benefits in enhancing surgical safety by providing real-time spinal cord function assessment, contributing to safer procedures and improved resection outcomes [65]. However, the lack of validated procedures for responding to intraoperative alerts represents a significant gap that future research should address [66].

## 7. Alternative Treatment

Currently, robotic surgery is primarily used for precise screw placement in cases of spinal instability [67] and in hybrid surgeries involving extensive tumors extending into other cavities, as demonstrated by Iga et al., who combined spinal endoscopy with robot-assisted intrathoracic surgery using the da Vinci robot for a dumbbell-shaped tumor [68].

Radiation therapy is a viable option for managing spinal cord neoplasms especially when facing unresectable lesions or residual disease post-surgery, or in cases where surgery is contraindicated due to medical comorbidities, performance status, lesion location, or rapidly recurring/progressive tumors [69]. Several studies have explored the use of stereotactic radiation therapy (SRT) or stereotactic radiosurgery (SRS) for the treatment of intradural extramedullary (IDEM) tumors, with generally positive outcomes and few transient complications reported.

Adjuvant chemotherapy plays a very limited role in benign spinal tumor treatment. Several studies evaluating various medical approaches aimed at inhibiting or stabilizing tumor growth were unable to show any clinical benefits for patients. Chemotherapy drugs may be utilized in the management of advanced or unresectable tumors, as shown specifically in [53]. Thus, adjuvant chemotherapy is not included in the standard therapy, regardless of the tumor’s location. The current literature lacks evidence supporting chemotherapy for benign glomus tumors, with no reported cases of malignant transformation or metastases in spinal canal glomangiomas. Hsieh et al. described a complex case involving uncertain malignancy and extensive penetration at the cervical level, requiring multiple rounds of radiotherapy [70]. In contrast, Hsieh reported no recurrence in a similar uncertain case, and Nagata documented long-term survival despite reoperation for a recurrent dumbbell-shaped tumor [71,72]. While glomus tumors in the spine have not progressed to malignancy, they have in other organs where systematic chemotherapy, particularly with agents like doxorubicin or ifosfamide, is advised despite unclear sensitivity. The PALETTE study found pazopanib effective in some sarcoma types but not in malignant glomus tumors [73,74]. Du et al. reported significant benefits from a genetically guided treatment combining Anlotinib and Tislelizumab for an esophageal MGT with metastases [74].

In summary, glomangiomas are uncommon soft tissue tumors, with spinal canal involvement being excitingly rare. Typically benign and slow-growing, similar to other tumors in this location, they predominantly present with local pain and neurological deficits resulting from spinal cord and nerve root compression. Despite limited documentation in the available literature, intradural glomangiomas have not been reported until now. However, the presented case highlights their potential presence within the dural sac. Regardless of location, complete surgical resection remains the preferred treatment, with minimally invasive approaches favored when feasible. Efforts are also underway to utilize robotic surgery or augmented reality to enhance surgical outcomes in spinal tumor treatment. Stereotactic surgery is a valuable option for cases where complete tumor removal is not possible or if the patient’s medical condition precludes surgery. Adjuvant chemotherapy or immunotherapy may be considered for tumors exhibiting malignant characteristics.

## Figures and Tables

**Table 1:** Histological classification of glomus tumors.

Glomus Tumors	
Benign	Malignant
Glomangioma	Tumors of uncertain malignant potential
Glomangiomyomas	Symplastic
	Glomangiomatosis

**Table 2:** Summary of spinal glomangiomas in the literature.

Author	Patient	Level	Symptom	Physical Examination	Time	Malignant Potential	Recurrence	Additional Treatment
E. Çetin et al.	65-year-old female	T7–T8. Dumbbell-shape.		Paresthesia and hyperreflexia in lower extremities. Loss of vibratory sense and moderate pinprick below T8	6 months	no	no	no
Z. Liao et al.	48-year-old male	The intervertebral foramen and T3 vertebral body. Tumor penetrated to thoracic cavity.	Chest discomfort with intermittent back pain.	No abnormalities in physical examination.	no data	no	no	no
C.-H. Kuo et al.	26-year-old male	T11. Epidural intraspinal.	Progressive paraparesis.	Progressive weakness and impaired sensation. Paresthesia and hyperreflexia in lower extremities. No sphincter symptoms.	3 months	no	no	no
X. Li et al.	47-year-old male	T7 to T9. Epidural intraspinal.	Unsteady gait.	Numbness of lower extremities especially on left side. Diminished sensation of pinprick and light touch below umbilicus Weakening of the myodynamics of both lower extremities.	5 months	no	no	no
Y. Li et al.	64-year-old female	The intervertebral foramen and epidural of C1–5.	Neck pain.	Numbness of the left side of body.	3 months	uncertain	After 4 months	radiotherapy
F. Babeau et al.	52-year-old female	The intervertebral foramen T6–T7.	Chronic back pain.	Palpation of T6 spinous process triggered the pain on physical examination.	3 months			
Hsieh et al.	37-year-old female	C7-T2 epidural space	Unsteady gait.	Progressive weakness and impaired sensation.	4 months	no	no	no
C. Axmann et al.	50-year-old male	The intervertebral foramen L1–L2	Chronic low back pain	No sensory impairments were observed. Moreover, the patient experienced muscle tightness as a result of persistent pain, and touching the lower back elicited discomfort.	no data	uncertain	no	no
current case	45-year-old male	Intradural, extramedullary, L3	Progressive gait impairment.	Diminished lower extremity tendon reflexes and mild weakness in knee extension and hip external rotation, devoid of sensory deficits	3 months	no	no	no

## Data Availability

Not applicable.
